# Group 2 innate lymphoid cells protect lung endothelial cells from pyroptosis in sepsis

**DOI:** 10.1038/s41419-018-0412-5

**Published:** 2018-03-06

**Authors:** Dengming Lai, Jing Tang, Linsong Chen, Erica K. Fan, Melanie J. Scott, Yuehua Li, Timothy R. Billiar, Mark A. Wilson, Xiangming Fang, Qiang Shu, Jie Fan

**Affiliations:** 1 Department of Thoracic and Cardiovascular Surgery, The Children’s Hospital of Zhejiang University School of Medicine, Hangzhou, Zhejiang China; 2 Department of Surgery, University of Pittsburgh School of Medicine, Pittsburgh, PA USA; 3 Department of Anesthesiology, Nanfang Hospital, Southern Medical University, Guangzhou, China; 4 Department of Thoracic Surgery, Shanghai Pulmonary Hospital, Tongji University School of Medicine, Shanghai, China; 5 University of Pittsburgh School of Arts and Science, Pittsburgh, PA USA; 6 Research and Development, Veterans Affairs Pittsburgh Healthcare System, Pittsburgh, PA USA; 7 McGowan Institute for Regenerative Medicine, University of Pittsburgh, Pittsburgh, PA USA; 8 Department of Anesthesiology and Intensive Care Unit, The First Affiliated Hospital, Zhejiang University School of Medicine, Hangzhou, Zhejiang China

## Abstract

Group 2 innate lymphoid cells (ILC2) are one of three subgroups of innate lymphoid cells (ILC1, ILC2, and ILC3), and the major ILC population detected in the lungs. The function of ILC2 in the regulation of lung inflammation remains unclear. In the current study, we explored an important role of ILC2 in protecting lung endothelial cell (EC) from pyroptosis in sepsis-induced acute lung inflammation and the underlying mechanism. Using a cecal ligation and puncture (CLP) mouse sepsis model, we demonstrated that IL-33, which is released in response to sepsis, acting through its receptor ST2 mediates ILC2 expansion in the lungs. We further showed that the increased ILC2 in the lungs secrete IL-9, which in turn prevents lung EC from undergoing pyroptosis, a pro-inflammatory cell death form, by attenuating caspase-1 activation. These findings suggest a previously unidentified innate pathway that negatively regulates lung inflammation following sepsis.

## Introduction

Sepsis is a life-threatening syndrome of organ dysfunction induced by dysregulated host responses to infection^1^. Although the hospital supportive care has been improved, sepsis is still a leading cause of mortality in the intensive care unit^2–4^. Mortality in sepsis is primarily due to multiple organ dysfunction syndrome, of which acute lung injury (ALI) is a common and important component^5^. At a cellular level, sepsis-induced endothelial dysfunction and injury of the pulmonary vasculature, especially the pulmonary microvasculature, are the predominant mechanisms for the development of ALI and are associated with higher mortality^6,7^.

Endothelial cells (ECs) serve as the sentinel cells for detecting infection and host defensing^8^. EC death triggered by lipopolysaccharide (LPS) and tumor necrosis factor α (TNFα) has been determined in septic mice^9,10^. Studies have shown that LPS through activating caspase-1 induces EC pyroptosis^11^, a caspase-1-dependent pro-inflammatory cell death type^12^. Cell pyroptosis, which can be triggered by various pathological stimuli, including infection, is characterized by plasma-membrane pore formation, intracellular content release, and DNA cleavage^12,13^. Lung EC pyroptosis plays a significant role in the progression of ALI^14^.

Innate lymphoid cells (ILCs), a new member of the lymphoid population, play a central role in innate immunity of host response to inflammation, infection, and tissue damage. ILCs are further divided into three subgroups. ILC1 include natural killer (NK) and ILC1 cells, which produce interferon-γ. ILC2 produce type 2 cytokines, e.g., interleukin (IL)-9 and IL-13, and are dependent on transcription factors GATA-binding protein 3 (GATA3) and retinoic acid receptor-related orphan receptor-α (ROR-α) for development and function. ILC3 include all ILC subtypes that produce IL-17 and/or IL-22, and their development and function depend on the transcription factor ROR-γt^15,16^. ILC2 have been identified in both human and mouse lungs and airways, and these cells serve as a main ILC subtype in the lungs. ILC2 are also known to play an important role in maintaining airway barrier integrity and lung tissue homeostasis after virus infection^16,17^, and so have been suggested to be protective during infection^17,18^. However, the role of ILC2, as a major ILC population in the lungs, remains poorly characterized during sepsis and sepsis-induced acute lung inflammation.

Studies have suggested that epithelial or myeloid cell-derived IL-25, IL-33, and thymic stromal lymphopoietin (TSLP) regulate ILC2 expansion and activation^17,19–22^. The expansion of tissue-resident ILC2 is followed by increased hematogenous emigration and redistribution of ILC2 under physiologic or pathological conditions^23^. Increased ILC2 in the lungs following sepsis was observed^24^. However, the mechanism driving ILC2 into the lungs in sepsis remains unclear.

This study aims to elucidate the mechanism of ILC2 expansion in the lungs following sepsis, and the role of ILC2 in the regulation of lung inflammation during sepsis. We demonstrate here that IL-33, which is released mainly by epithelial cells in response to sepsis, acting through its receptor ST2 induces ILC2 expansion in the lungs. Increased ILC2 in the lungs secrete IL-9, which in turn prevents lung EC from undergoing pyroptosis by attenuating caspase-1 activation. These findings suggest a previously unidentified pathway that negatively regulates lung inflammation following sepsis.

## Results

### Sepsis induces ILC2 expansion in the lungs and peritoneal cavity

**Fig. 1:**
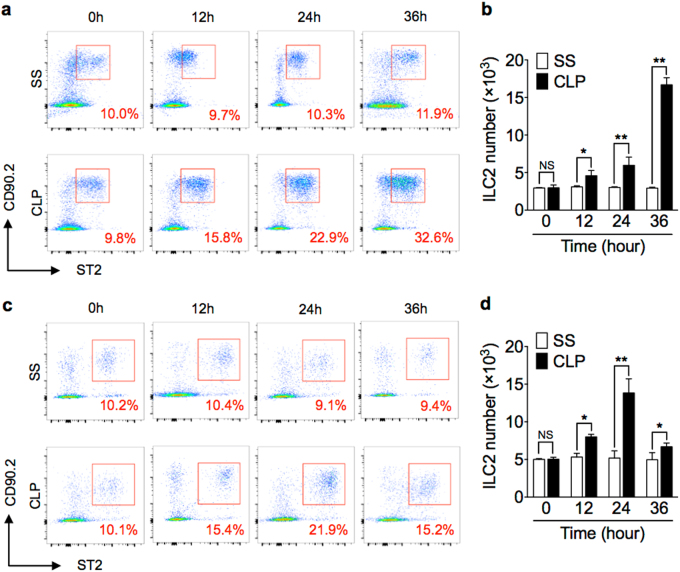
Sepsis induces ILC2 expansion in lungs and peritoneal cavity. Representative flow cytometry plots showing percentages of Lin^−^CD45^+^CD90.2^+^ST2^+^ ILC2 in **a** lung and **c** peritoneal lavage fluid of WT mice at 0, 12, 24, and 36 h after CLP or sham surgery (SS). Bar graphs showing ILC2 absolute cell number in **b** lung and **d** peritoneal cavity in SS or CLP mice at time points up to 36 h. *n* = 6 mice/group. Data are shown as mean ± SEM. *P < 0.05, **P < 0.01, NS not significant

Sepsis was induced in wild-type (WT) mice using a clinically relevant model of polymicrobial peritonitis caused by cecal ligation and puncture (CLP)^25^. ILC2 in the lungs at baseline and time points up to 36 h after CLP or sham surgery (SS) were detected by flow cytometry and defined as Lin^−^CD45^+^CD90.2^+^ST2^+^ cells (see gating strategy in Supplementary Fig. 1a)^26^. Additional ILC2 markers, including Sca-1, KLRG1, CD25, and CD127 were also used to further confirm ILC2 identification (Supplementary Fig. 1b)^17,26^. The percentage and absolute number of ILC2 in the lungs at 12 h after CLP was significantly increased as compared with that in SS mice, and the ILC2 number remained elevated for at least additional 24 h (Fig. 1a, b). The percentage and absolute number of ILC2 in the lungs of SS mice did not show significant changes.

ILC2 in the peritoneal lavage fluid (PLF) were also measured after CLP. As shown in Fig. 1c, d, CLP induced an increase in the percentage and absolute number of peritoneal ILC2, which peaked at 24 h, but remained significantly elevated at 36 h after CLP.

These data indicate that ILC2 increase in the lungs and peritoneal cavity in response to CLP-induced sepsis.

### IL-33/ST2 signaling is required for sepsis-induced ILC2 expansion

**Fig. 2:**
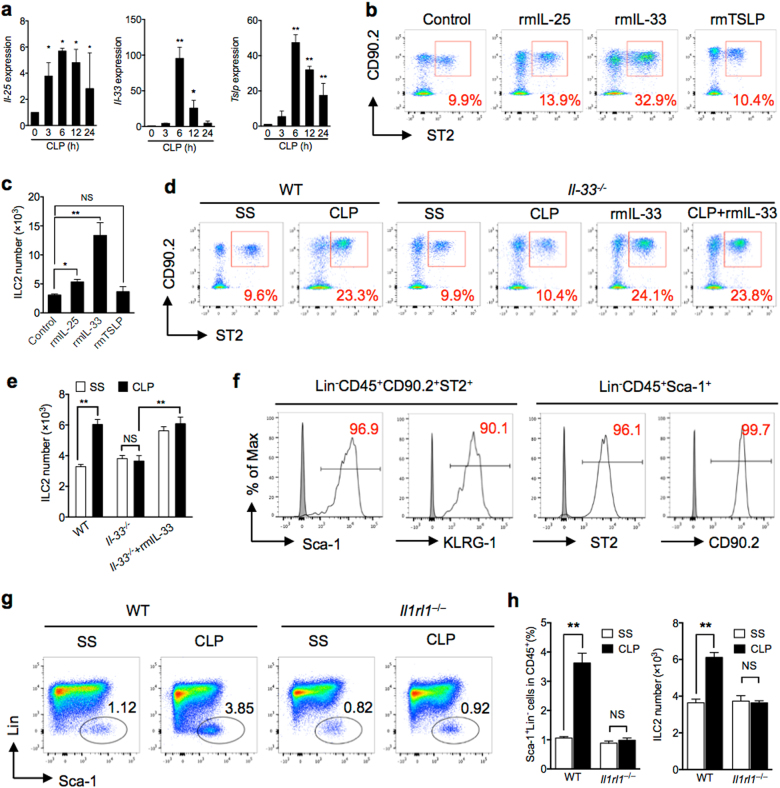
IL-33/ST2 signaling is required for sepsis-induced ILC2 recruitment. **a** RT-qPCR expression of *Il-25*, *Il-33*, and *Tslp* mRNA in lung tissue, which are relative to 18 s, at time points up to 24 h after CLP compared to SS of each time points (*n* = 3–5 mice/group). **b** Representative FACS plots and **c** bar graph showing absolute number of ILC2 in lungs harvested from control (PBS), rmIL-25, rmIL-33, and rmTSLP-treated mice (1 μg in 50 μl PBS intratracheal (*n* = 5 mice/group). **d** Representative flow cytometry plots and **e** absolute number of lung ILC2 (Lin^−^CD45^+^CD90.2^+^ST2^+^) in WT and *Il-33*^*−/−*^ mice with/without rmIL-33 treatment mice at 24 h after CLP or SS. Data are representative of three experiments, *n* = 3–6. **f** Expression of ILC2-identifying cell surface markers (Sca-1 and KLRG1) on Lin^−^CD45^+^CD90.2^+^ST2^+^ cells, and expression of ST2 and CD90.2 on Lin^−^CD45^+^Sca-1^+^ cells. **g** Bar graphs showing representative FACS plots and **h** absolute cell number of lung ILC2 in WT or *Il1rl1*^*−/−*^ mice as above (*n* = 5 mice/group). The plots were gated at CD45^+^ cells. Data shown as mean ± SEM. *P < 0.05, **P < 0.01, NS not significant

TSLP, IL-25, and IL-33 were previously suggested to play a role in mediating ILC2 expansion and activation^17,19–22^. We therefore measured *Tslp*, *Il-25*, and *Il-33* mRNA expression, using Real-time PCR, in the lungs of CLP mice for up to 24 h; and found that the expression of all these cytokines were significant increased and peaked at 6 h after CLP (Fig. 2a). To determine whether these cytokines mediate sepsis-induced ILC2 recruitment in the lungs, we intratracheally (i.t.) injected normal WT mice with recombinant mouse TSLP (rmTSLP), IL-25 (rmIL-25), IL-33 (rmIL-33), or phosphate-buffered saline (PBS; control), and assessed percentage and absolute number of ILC2 in the lungs at 24 h after the injection. Treatment with rmIL-33 induced significant increase of ILC2 in the lungs as compared with PBS control group (Fig. 2b, c). It is noticeable that the number of ILC2 in the lungs induced by IL-33 is similar to that induced by CLP at 24 h. Treatment with rmIL-25 also increased the percentage and number of ILC2 in the lungs as compared with PBS-treated group, but the levels were significantly lower than that in the rmIL-33-treated mice and CLP mice (Fig. 2b, c). Despite the increase in *Tslp* mRNA expression in the lungs after CLP, treatment with rmTSLP did not increase the percentage and number of ILC2 in the lungs as compared with PBS-treated group (Fig. 2b, c).

To confirm the role of IL-33 in mediating ILC2 expansion in the lungs, we first detected IL-33 receptor ST2 expression on the ILC2. Since the Sca-1 and KLRG-1 double-positive in lineage-negative cells were nearly 100% GATA3-positive and has been defined as ILC2^27^, we used the strategies by measuring the cell markers of Sca-1 and KLRG-1 on Lin^−^CD45^+^CD90.2^+^ST2^+^ cells and the expression of ST2 and CD90.2 on Lin^−^CD45^+^Sca-1^+^ cells to define the ST2 expression on ILC2. We found that more than 90% of Lin^−^CD45^+^CD90.2^+^ST2^+^ cells express Sca-1 and KLRG-1, and ~99% of Lin^−^CD45^+^Sca-1^+^ cells express ST2 and CD90.2 (Fig. 2f). We then subjected *Il-33*^*−/−*^ mice and *Il1rl1*^*−/−*^ mice (genetic deficiency of ST2) to CLP and measured the percentage and number of Lin^−^CD45^+^Sca-1^+^ cells, which were also defined as ILC2^24^, in the lungs at 24 h after CLP. We demonstrated that deficiency of either *Il-33* or *Il1rl1* prevented ILC2 expansion in the lungs following sepsis (Fig. 2d, e, g, h). Moreover, i.t. administration of rmIL-33 to *Il-33*^*−/−*^ mice restored the increase of ILC2 in the lungs at 24 h after CLP and even SS (Fig. 2d, e). Collectively, these results strongly indicate a role for IL-33/ST2 signaling in ILC2 expansion in the lungs following sepsis.

### ILC2 protect lung EC from death following sepsis

**Fig. 3:**
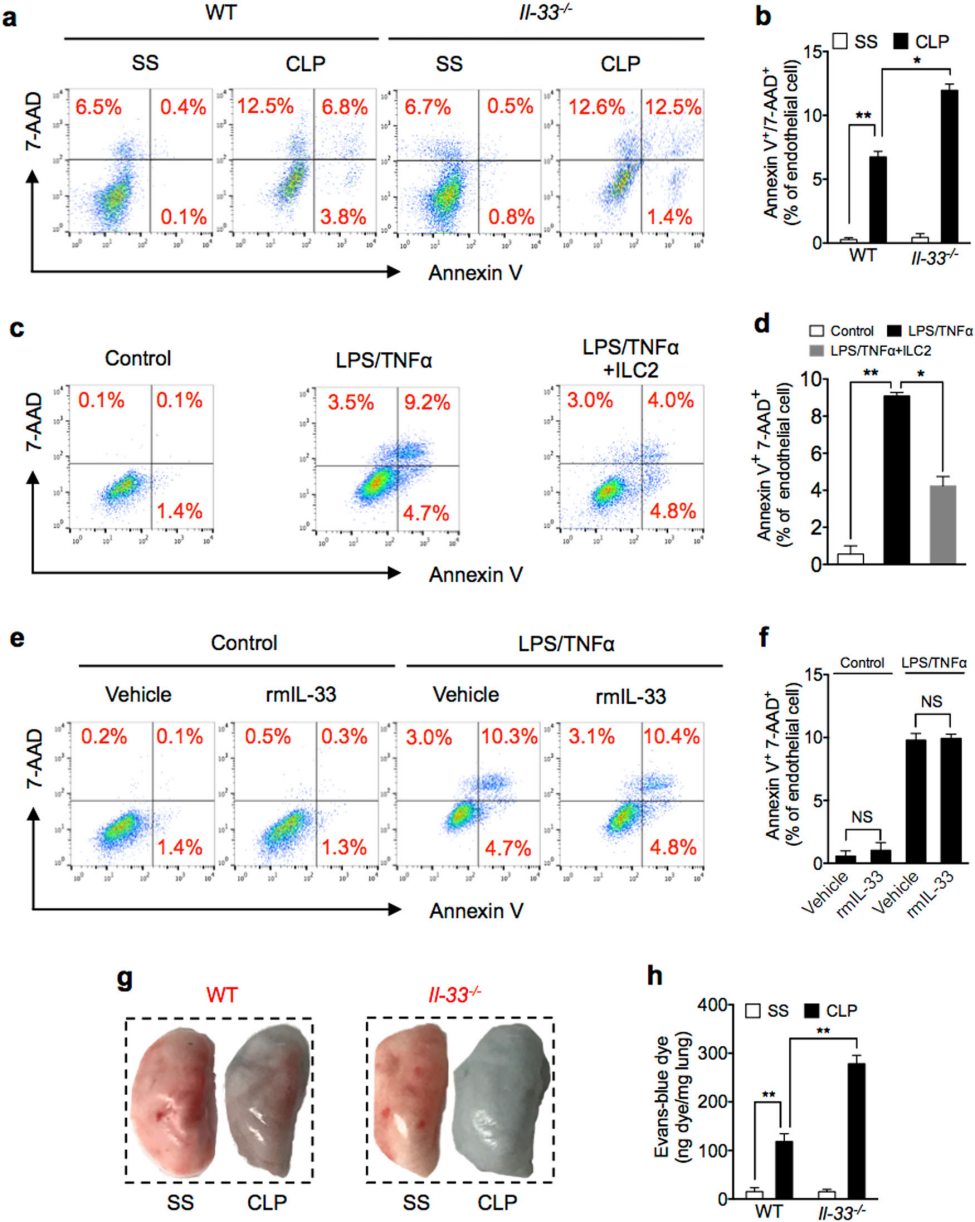
ILC2 protect lung EC from death following sepsis. **a** Representative flow cytometry plots of Annexin V/7-AAD staining of MLEC from WT and *Il-33*^*−/−*^ mice at 24 h after SS or CLP. MLECs were identified as CD31^+^ and Annexin V/7-AAD double-stained cells were analyzed as dying (*n* = 5 mice/group). **b** Bar graph of Annexin V/7-AAD double-stained cells as a percentage of MLEC in WT and *Il-33*^*−/−*^ mice at 24 h after SS or CLP. **c** Representative flow cytometry plots of Annexin V/7-AAD staining of in vitro primary isolated MLEC from WT mice cultured alone (control), with LPS (1 μg/ml) + TNFα (20 ng/ml), or LPS + TNFα + co-culture with ILC2 (1 × 10^4^ cells/well) for 24 h. **d** Bar graph of Annexin V/7-AAD double-stained cells as a percentage of total MLEC. **e** Representative flow cytometry plots and **f** bar graph of Annexin V/7-AAD staining of purified MLEC from WT mice cultured with LPS (1 μg/ml) + TNFα (20 ng/ml) and with or without rmIL-33 (50 ng/ml) for 24 h. **g** Representative images of whole lungs stained with Evans blue dye from WT and *Il-33*^*−/−*^ mice at 24 h following SS or CLP. **h** Colorimetric quantitative analysis of Evans blue dye extracted from stained lungs from WT and *Il-33*^*−/−*^ mice at 24 h following SS or CLP (*n* = 5 mice/group). Data are representative of three independent in vitro experiments. Data shown are the mean ± SEM. *P < 0.05, **P < 0.01

EC death plays a significant role in pulmonary microvascular dysfunction associated with ALI following sepsis^9^. Lung ILC2 have been suggested to play a role in restoring airway integrity and lung tissue homeostasis^17^. These findings, therefore, led us to ask whether lung ILC2 involve in maintaining the integrity of lung EC in sepsis. We subjected WT and *Il-33*^*−/−*^ mice to CLP and measured mouse lung EC (MLEC) death using Annexin V/7-AAD staining detected by flow cytometry. As shown in Fig. 3a, b, CLP markedly induced MLEC death in *Il-33*^*−/−*^ mice as compared with that in WT mice.

To determine a causal relationship between the decreased ILC2 in the lungs and increased MLEC death in *Il-33*^*−/−*^ mice following sepsis, we co-cultured ILC2 with mouse MLEC in vitro in the presence and absence of LPS and TNFα, which mimic sepsis stimulation. As shown in Fig. 3c, d, co-culture with ILC2 markedly reduced MLEC death induced by LPS and TNFα, suggesting a direct protective effect of ILC2 on MLEC in sepsis. However, addition of rmIL-33 in the culture medium failed to protect MLEC death (Fig. 3e, f).

To further determine the impact of MLEC death on lung vascular leaking, we applied Evans blue dye (EBD) to assess blood vessel permeability in vivo. We demonstrated that at 24 h after CLP, the lungs of *Il-33*^*−/−*^ mice had increased permeability shown as more blue coloration in the lungs than that in WT lungs (Fig. 3g). The EBD extravasation was also quantified. As shown in Fig. 3h, EBD concentration was twofold higher in the lungs of *Il-33*^*−/−*^ mice than that in WT mice.

These data demonstrated that IL-33-mediated ILC2 expansion in the lung prevents MLEC from death, but not IL-33 itself.

### Characterization of cytokine expression and secretion from lung ILC2 in sepsis

**Fig. 4:**
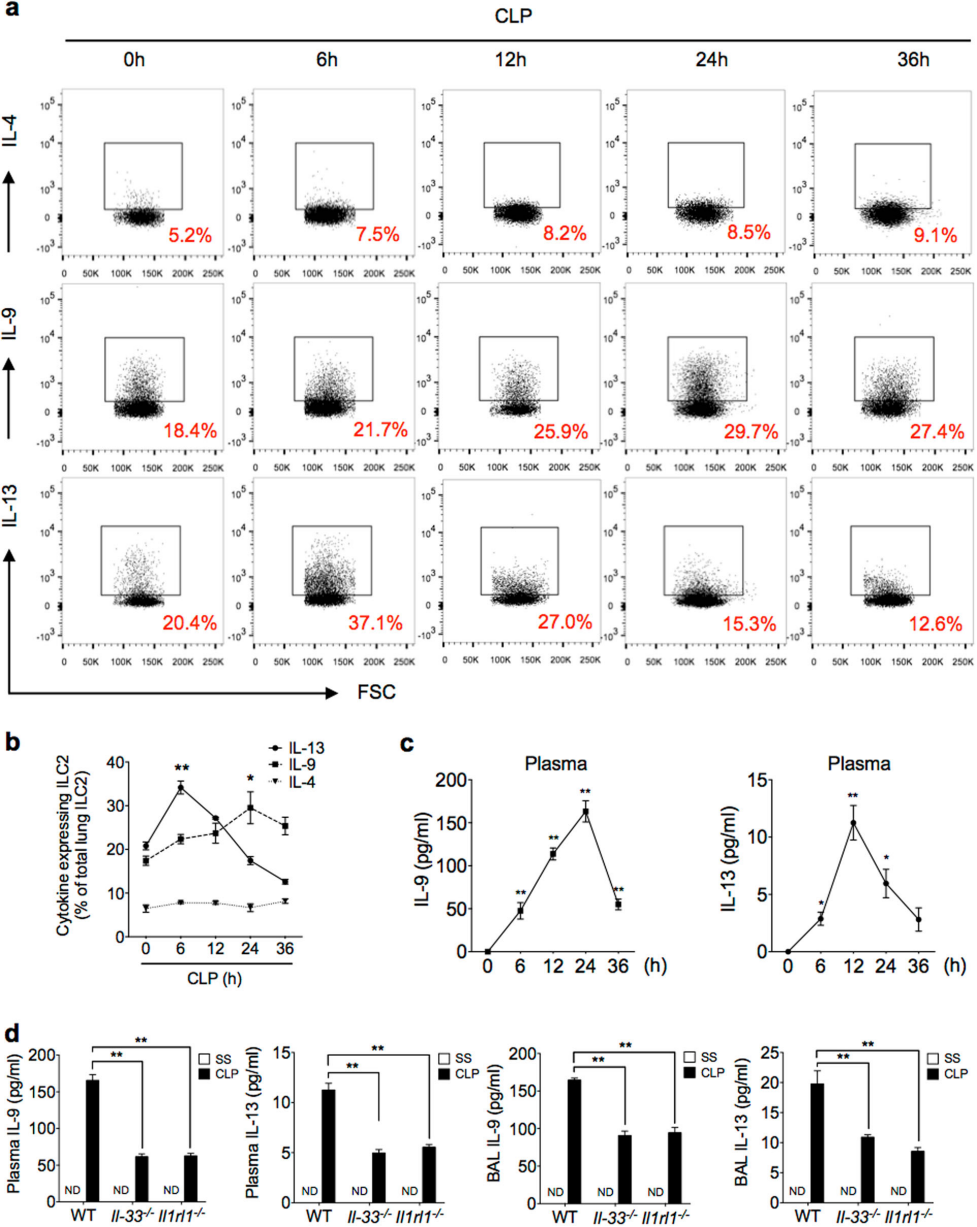
Characterization of cytokine expression and secretion from lung ILC2 in sepsis. **a**, **b** Representative flow cytometry plots and graph of quantitation of percentages of IL-4^+^, IL-9^+^, and IL-13^+^ lung ILC2 from WT mice collected at time points up to 36 h after CLP or SS and stimulated with PMA (50 ng/ml), ionomycin (500 ng/ml), and brefeldin A (1 μg/ml) for 4 h prior to intracellular cytokine determination by flow cytometry. *n* = 5–8 mice/group. **c** Plasma IL-9 and IL-13 concentration in WT mice measured by ELISA at time points up to 36 h after CLP or SS. **d** Plasma and bronchoalveolar lavage fluid (BALF) levels of IL-9 at 24 h and IL-13 at 12 h in WT, *Il-33*^*−/−*^, and *Il1rl1*^*−/−*^ mice after SS or CLP determined by ELISA (*n* = 5 mice/group). Data shown are the mean ± SEM. *P < 0.05, **P < 0.01

Studies have shown that ILC2 are an important cellular source of type 2 cytokines^17,19,28^. To characterize the type 2 cytokines secreted from lung ILC2 following sepsis, we measured IL-4, IL-9, and IL-13 expression in the ILC2 increased in the lungs by flow cytometry. We found that IL-9 positive (IL-9^+^) ILC2 significantly increased by 24 h after CLP, and IL-13^+^ ILC2 transiently increased after CLP and reached a peak at 6 h (Fig. 4a, b). IL-4^+^ ILC2, however, did not significantly increase following sepsis (Fig. 4a, b). We then measured plasma concentrations of IL-9 and IL-13 following sepsis, and demonstrated that both cytokines were markedly increased between 12 and 24 h after CLP (Fig. 4c). However, both *Il-33*^*−/−*^ and *Il1rl1*^*−/−*^ mice exhibited reduced levels of IL-9 and IL-13 in plasma and bronchoalveolar lavage fluid (BALF) after sepsis as compared with those in WT mice (Fig. 4d). These results suggest a specific pattern of cytokine expression and secretion from lung ILC2, which increased in the lungs following sepsis in an IL-33/ST2-dependent manner.

### ILC2-derived IL-9 protects MLEC from pyroptosis

**Fig. 5:**
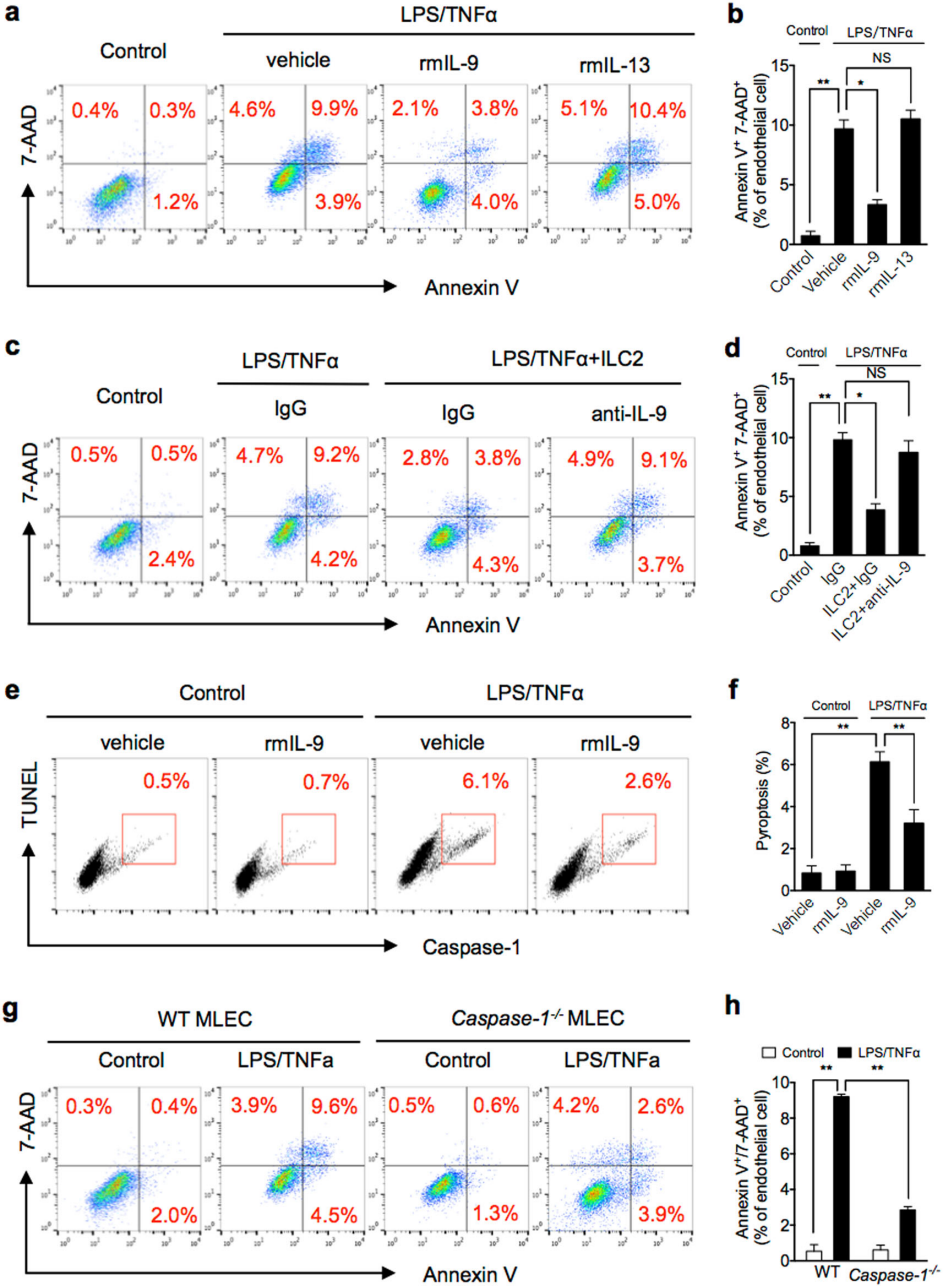
ILC2-derived IL-9 protects MLEC from pyroptosis. **a**, **b** Representative flow cytometry plots and quantitation of Annexin V/7-AAD staining of MLEC cultured with LPS/TNFα and with or without rmIL-9 or rmIL-13 for 24 h. **c**, **d** Representative flow cytometry plots and quantitation of Annexin V/7-AAD staining of MLEC co-cultured with ILC2 (1 × 10^4^ cells/well) and LPS (1 μg/ml) + TNFα (20 ng/ml) and with IgG or anti-IL-9 for 24 h. **e**,** f** Representative flow cytometry plots and quantitation of MLEC pyroptosis (caspase-1/TUNEL double-positive cells) with rmIL-9 (50 ng/ml), LPS, and TNFα for 24 h. **g**, **h** Representative flow cytometry plots and quantitation of Annexin V/7-AAD staining of MLEC from WT or *Caspase-1*^*−/−*^ mice with LPS/TNFα for 24 h. MLECs were identified as CD31^+^ cells. Data shown are the mean ± SEM, *n* = 3–6 mice/group. *P < 0.05, **P < 0.01

IL-9 has recently been reported as a regulator in the pathogenesis of many inflammatory diseases^29,30^. For example, IL-9 is involved in protective immunity to *Trichuris muris* infections^31^, promotes Treg activation and prevents excessive cartilage destruction and bone loss in arthritis^32^, and promotes tissue repair by amplifying ILC2 function in helminth-induced lung inflammation^33^. In lung tissue, IL-9 production was restricted to IL-33-induced ILC2 in physiological or inflammatory conditions^34^. IL-9 induces vascular endothelial adhesion molecule-1 expression on mouse aortic EC and attenuates lung inflammation^29,33,35^. We hypothesized that IL-9 might mediate the ILC2-dependent MLEC protection in sepsis. To test this hypothesis, we first determined whether MLECs express cell surface IL-9 receptor (IL-9R). As shown in Supplementary Fig. 2a, MLECs express a considerable level of IL-9R. We then treated MLECs with LPS and TNFα in vitro for 24 h in the presence and absence of rmIL-9 or rmIL-13, and found that rmIL-9 significantly reduced MLEC death; whereas, rmIL-13 failed to protect the MLEC from death (Fig. 5a, b). Furthermore, neutralizing antibody against IL-9 was added into the ILC2-MLEC co-culture system, which challenged with LPS and TNFα, and demonstrated that ILC2 no longer protect the MLEC from death (Fig. 5c, d).

We further defined the MLEC death type by staining the cells with TUNEL and Alexa Fluor 488-labeled caspase-1 FLICA following LPS and TNFα challenge. We found that TUNEL and activated caspase-1 double-positive MLECs, which represent pyroptosis, occupied ~70% of the MLEC death (Fig. 5e, f), and IL-9 reduced MLEC pyroptosis by ~60% (Fig. 5e, f).

To further confirm the observed MLEC death is dependent on caspase-1 activation, we treated MLEC from *Caspase-1*^*−/−*^ mice with LPS and TNFα for 24 h and measured the level of cell death. Figure 5g, h shows that caspase-1 deficiency reduced MLEC death by ~70%.

Taken together, these results suggest that ILC2-derived IL-9 mediates the protection of MLEC from pyroptosis.

### IL-9 decreases caspase-1 activation in lung EC

**Fig. 6:**
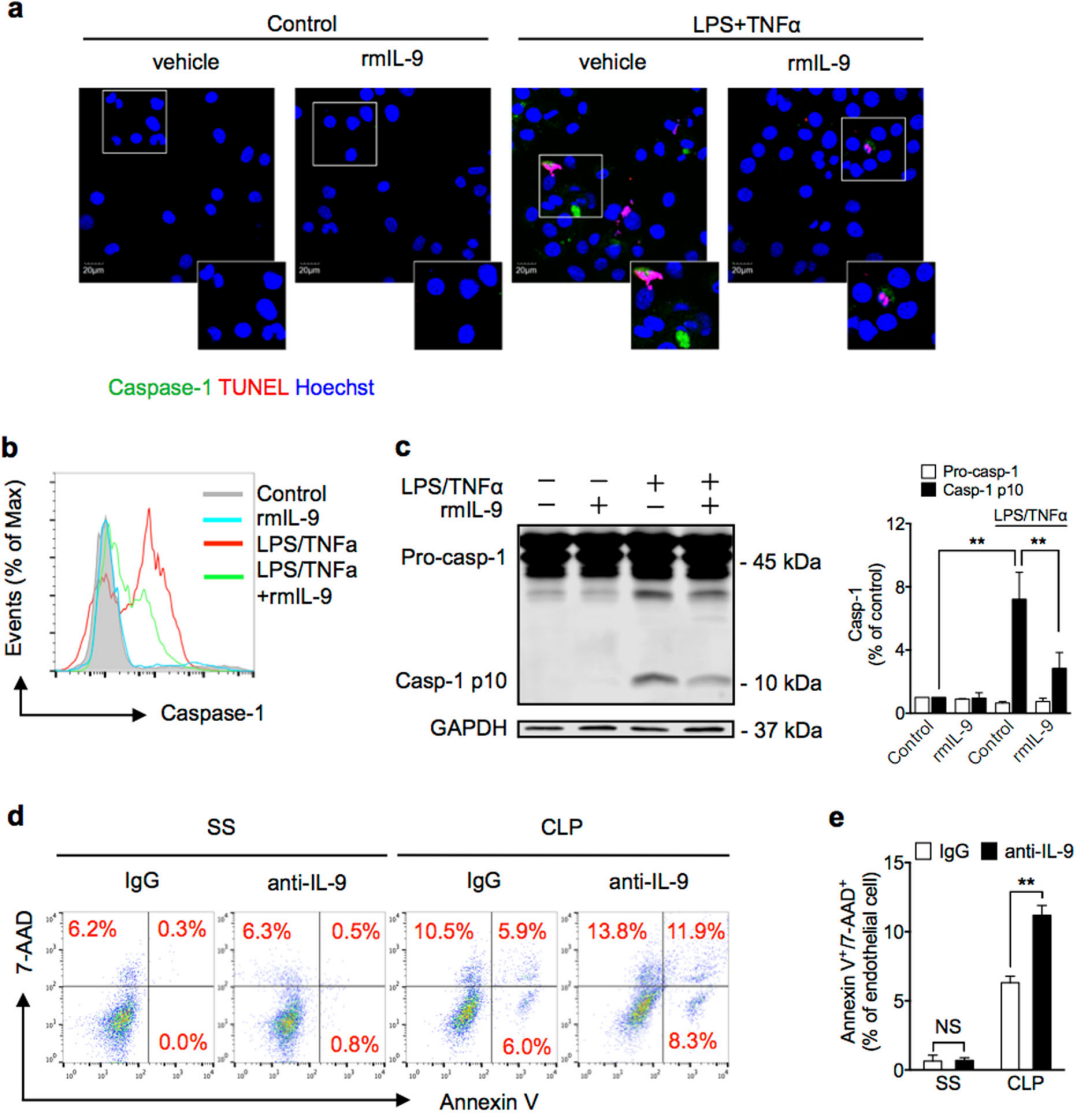
IL-9 decreases caspase-1 activation in lung EC. **a** Confocal microscopy immunofluorescence of MLEC stained for activated caspase-1 (green), TUNEL (red), and Hoescht (blue) at 24 h cultured with rmIL-9 (50 ng/ml), LPS, and TNFα. **b** Number of MLEC expressing activated caspase-1 at 6 h after treatment with LPS and TNFα with or without rmIL-9 (50 ng/ml). **c** Western blots of pro-caspase-1 (Pro-casp-1) and activated/cleaved caspase-1 (Casp-1 p10) in whole-cell lysates of MLEC at 6 h after LPS, TNFα, and/or rmIL-9 (50 ng/ml). **d** Representative flow cytometry plots of MLEC from WT mice treated with nonspecific IgG or anti-IL-9 antibody (1 mg/kg) at 30 min before SS or CLP. MLECs were isolated at 24 h after CLP, and identified as CD31^+^, and Annexin V/7-AAD double-stained cells were analyzed as dying (*n* = 5 mice/group). **e** Bar graph of Annexin V/7-AAD double-stained cells as demonstrated in **d**. Data shown are the mean ± SEM, *n* = 3–6 mice/group in vivo. *P < 0.05, **P < 0.01

To determine whether IL-9 attenuates MLEC pyroptosis through suppressing caspase-1 activation, we treated the MLEC with LPS and TNFα for 24 h in the presence and absence of rmIL-9, and visualized caspase-1 activation and nuclear fragmentation by staining the cells with Alexa Fluor 488-labeled caspase-1 FLICA and TUNEL in the MLEC and observed using confocal microscopy. As shown in Fig. 6a, LPS and TNFα induced caspase-1 activation and nuclear fragmentation in the MLEC, whereas rmIL-9 decreased caspase-1 activation in the MLEC. Intracellular caspase-1 activation was also measured in the MLEC by flow cytometry and western blotting. IL-9 exhibited a significant suppressive effect on caspase-1 activation in the MLEC in response to LPS and TNFα (Fig. 6b, c). To confirm the protective role of IL-9 in CLP-induced MLEC death, we injected the CLP mice (i.t.) with neutralizing antibody against IL-9, and found that the IL-9 antibody increased MLEC death following CLP (Fig. 6d, e). Altogether, these data show a novel role for ILC2-derived IL-9 in protection of MLEC from pyroptosis through suppressing caspase-1 activation.

## Discussion

A number of studies have identified ILC2 in lung tissue and implicated these cells in the regulation of immunity and inflammation in the lung^17^. The data presented here show that IL-33/ST2 signaling leads to a significant expansion of ILC2 cells in the lungs and peritoneal cavity following CLP-induced sepsis. Expansion of ILC2 in the lungs provides ILC2-derived IL-9 in the lungs, which reduces sepsis-induced EC pyroptosis through suppressing caspase-1 activation. These findings represent a novel pathway that regulates lung EC death and thereby affecting the progression of acute lung inflammation following sepsis.

Epithelial, endothelial, and myeloid cells in most organs express IL-25, IL-33, and TSLP, these are known to modulate ILC2 expansion in the lungs and activation in inflammation^17,19–22,36^. In the current study, we show that IL-25, IL-33, and TSLP expression increased in response to systemic inflammation caused by experimental peritonitis, and sepsis induces expansion of ILC2 in the lungs consistent with the changes in the expression of IL-25, IL-33, and TSLP. However, it was only the absence of IL-33, and its receptor ST2, that the increase in lung ILC2 cells was abolished and type 2 cytokines, including IL-9 and IL-13 were decreased, and rmIL-33 could rescue the phenotype, highlighting a role for IL-33 in ILC2 expansion and activation^22^.

EC death has been reported to play an important role in activation of a number of inflammatory cells, release of inflammatory mediators, and induction of inflammation in sepsis^37^. Previous report showed that EC death occurs in sepsis^38^, and TNF superfamily-associated pathways and activated caspases mediate the endothelial death^10,39^. We also observed lung EC death, as defined with the positive markers of CD31, Annexin V, and 7-AAD, following sepsis. Recently, IL-9 has been reported to be involved, either beneficially or deleteriously, in the pathogenesis of some diseases related to inflammation^29,30^. ILC2 is the main source of IL-9 in mouse lung tissue in physiological or inflammatory circumstances^24^. Our data showed that genetic deficiency of *Il-33* prevented ILC2 expansion in the lung and increased MLEC death following CLP-induced sepsis; and addition of IL-33 into MLEC did not affect MLEC death in response to LPS and TNFα. However, co-culture of MLEC with ILC2 significantly decreased MLEC death. Meanwhile, neutralization of IL-9 with anti-IL-9 antibody diminished the beneficial effect of ILC2. Furthermore, restoring IL-9 by addition of IL-9 into the co-cultures decreased the LPS/TNFα-induced MLEC death. These results indicate a protective role for ILC2-derived IL-9 in protecting lung EC from death following sepsis.

Cell death plays a significant role in inflammation and infection. The types of cell death can be non-programmed or programmed. Programmed cell death includes apoptosis, autophagy, necrosis, necroptosis, and pyroptosis. Pyroptosis is a caspase-1-dependent form of cell death^12^, which characterized by pore formation in the plasma membrane, cell swelling, and DNA cleavage^40^. Thus, pyroptotic cell should be Annexin V and propidium iodide double-positive^41^. In our study, we have observed Annexin V^+^7-AAD^+^ EC in lung after CLP. And then we determined that IL-9 protects EC from pyroptosis in vitro by caspase-1 and TUNEL staining. The death of *Caspase-1*^*−/−*^ MLEC was reduced by ~70%, as compared with WT MLEC, indicating that caspase-1-dependent pyroptosis serves as a major form of MLEC death following sepsis. To address the mechanism of IL-9 protect EC from pyroptosis, we determined the caspase-1 activation. ILC2-derived IL-9, as shown in the data, through attenuating caspase-1 activation in MLEC reduces MLEC pyroptosis in sepsis.

In summary, the current study reveals a previously unidentified mechanism by which innate pathway negatively regulating lung inflammation following sepsis. The mechanism includes the regulation of enhanced ILC2 expansion in the lung and the protective role of ILC2-derived IL-9 in reducing lung EC pyroptosis.

## Materials and methods

### Mice

Male C57BL/6J WT mice were purchased from Jackson Laboratories (Bar Harbor, ME). *Il-33*-deficient (*Il-33*^*−/−*^) and *Il1rl1*-deficient (*Il1rl1*^*−/−*^, deficiency of ST2) mice were bred and maintained under specific pathogen-free conditions at the Animal Facility of the University of Pittsburgh School of Medicine. All mice were on a C57BL/6 genetic background and were carried out with 8-week-old male mice according to the guidelines of the Institutional Animal Care and Use Committee of the University of Pittsburgh, VA Pittsburgh Healthcare System.

### CLP model

Sepsis was induced by CLP model as described previously^25^. Briefly, mice were anaesthetized by intraperitoneal administration of ketamine (50 mg/kg) and xylazine (5 mg/kg). A double puncture was made through the cecum with an 18-gauge needle. A small amount of feces was gently squeezed out of the perforation sites to ensure patency of punctures. The cecum was then relocated into the abdominal cavity, and the laparotomy was closed. Mice received a subcutaneous injection of 1 ml PBS immediately after surgery for fluid resuscitation, and then were returned to their home cages. At the study end point, mice were sacrificed, BALF, PLF, and lung tissue were collected under sterile conditions, and blood was withdrawn by cardiac puncture.

### Treatment of mice

Mice were treated i.t. with 1 μg recombinant murine IL-33 (BioLegend, San Diego, CA) or IL-25 and TSLP (R&D Systems, Minneapolis, MN) in a volume of 50 μl PBS/mouse for 24 h. Anti-IL-9 antibody (R&D Systems, 1 mg/kg, i.t.) or a control antibody IgG was injected 30 min before SS or CLP surgery.

### ILC2 isolation and sorting from lung tissue

ILC2 isolation and sorting from lung tissue was performed as described previously^27^. Briefly, lungs were perfused with 10 ml PBS through right ventricle of heart, and then filled with 1 ml RPMI medium with Liberase TM (50 μg/ml final concentration) and DNase I (1 μg/ml final concentration) and digested in 5 ml RPMI digestion medium for 30–45 min at 37 °C with vortexing every 10 min. The resultant samples were mashed by 70 μm cell strainers, washed with Dulbecco’s modified Eagle media [DMEM; supplemented with 10% fetal bovine serum (FBS) and 1% penicillin/streptomycin (Thermo Fisher Scientific, Pittsburgh, PA)], and the remaining red blood cells were lysed. Cell suspensions were used for subsequent flow cytometry staining. For ILC2 sorting, total lung cells were stained with lineage cocktail Abs, against B220, CD3, CD4, CD5, CD8α, CD11b, CD11c, CD19, Gr-1, TCRβ, Ter-119, γδTCR, NK1.1, and FcεR1 (eBioscience, San Diego, CA); anti-CD45 Ab (eBioscience); anti-ST2 Ab (MD Biosciences, Oakdale, MN); and anti-CD90.2 Ab (eBioscience), at 4 °C for 30 min. ILC2 were defined as Lin^−^CD90.2^+^CD45^+^ST2^+^ and sorted by FACSAria (BD Biosciences). The average purity of ILC2 is >98%.

### MLEC isolation and culture

MLECs were isolated as described previously^42^. Briefly, mice were anesthetized, chest cavity was opened, and blood was removed by infusing 10 ml PBS. Lung tissues were diced into ~1 mm^3^ pieces and cultured in a 60 mm culture dish in growth medium (DMEM containing 2 mM glutamine, 10% FBS, 5% human serum, 50 μg/ml penicillin/streptomycin, 5 μg/ml heparin, and 80 μg/ml EC growth supplement from bovine brain) at 37 °C with 5% CO_2_ for 60 h. Then, remove tissue dices from dish and culture the adherent cells for 3 days. Purify the MLEC by biotin-conjugated rat anti-mouse CD31 mAb and BD IMag streptavidin particles plus-DM, and the immunomagnetic separation system (BD Biosciences) following the manufacturer’s instructions. Purified MLECs were characterized by their cobblestone morphology.

### Lung permeability assay

Lung vascular permeability was measured by EBD leakage in lung alveolus^43,44^. EBD (20 mg/kg; Sigma-Aldrich, St. Louis, MO) in 100 μl volume was administered intravenously through tail vein 1 h before animal euthanasia. Lungs were then perfused with PBS through right ventricle to remove intravascular dye. Remove and photograph the lungs. Then the lungs were dried at 60 °C for 48 h and incubated in formamide (Sigma-Aldrich) at 37 °C for 24 h and centrifuged at 5000 × *g* for 30 min. EBD in the supernatant was measured by spectrophotometric method (wavelength 620 and 740 nm). The extravasated EBD concentration was calculated according to a standard curve and expressed as the dye incorporated per mg of tissue.

### Flow cytometry

Cell suspensions were stained with a combination of the following monoclonal fluorescently conjugated antibodies: fluorescein isothiocyanate-conjugated B220 (RA3-6B2, eBioscience), CD3 (17A2, eBioscience), CD4 (RM4-5, eBioscience), CD5 (53-7.3, eBioscience), CD8α (53-6.7, eBioscience), CD11b (M1/70, eBioscience), CD11c (N418, eBioscience), CD19 (eBio1D3, eBioscience), Gr-1 (RB6-8C5, eBioscience), TCRβ (N57-597, eBioscience), Ter-119 (Ter119, eBioscience), γδTCR (eBioGL3, eBioscience), NK1.1 (PK136, eBioscience), and FcεR1 (MAR-1, eBioscience); phycoerythrin (PE)-conjugated ST2 (DJ8, MD Biosciences); allophycocyanin-conjugated CD90.2 (53-2.1, eBioscience); Alexa Fluor 700-conjugated CD45 (30-F11, eBioscience); PE-Cyanine 7-conjugated KLRG1 (2F1, BD Biosciences); and BV421-conjugated-Ly-6A/E (D7, BD Biosciences). To stain for intracellular murine antigens, cells were first stained for surface antigens, then fixed and permeabilized with intracellular fixation and permeabilization buffer set (eBioscience), according to the manufacturer’s recommendations. Cells were then incubated with specific antibodies to IL-4-PE-Cyanine 7 (11B11, eBioscience), IL-9-Percp-Cy5.5 (D9302C12, BD Biosciences), and IL-13-eFluor 450 (eBio13A, eBioscience). For intracellular staining, cells were incubated with Phorbol 12-myristate 13-acetate (PMA) (50 ng/ml), ionomycin (500 ng/ml) and brefeldin A (1 μg/ml) for 4 h. Cells were then washed in PBS and re-suspended. Cells were analyzed by BD LSRII flow cytometer (BD Bioscences) and FlowJo software.

### RNA isolation and real-time quantitative PCR

Total RNA from lung tissue was isolated by TRIZOL reagent (Life Technologies, Pittsburgh, PA) according to the manufacturer’s instructions. cDNA was generated using iScript Reverse Transcription Supermix (Bio-Rad, Hercules, CA). Quantitative real-time PCR was performed using SYBR Green Supermix (Bio-Rad) and run in a Bio-Rad iQ5 real-time PCR machine. All data were normalized to 18 s values, analyzed using comparative CT method and presented as the fold-increase over WT controls.

### Enzyme-linked immunosorbent assay

Cytokine and chemokine concentrations in plasma, BALF, and PLF were determined by IL-9 and IL-13 enzyme-linked immunosorbent assay (eBioscience) according to each manufacturer’s instructions.

### MLEC immunofluorescence staining

For cell death measurement, purified MLECs were stained with Alexa Fluor 488-labeled FLICA (ImmunoChemistry, Bloomington, MN), and TUNEL staining (Roche Applied Science, Indianapolis, IN), nuclear staining with 1 μg/ml Hoechst 33258 (Sigma-Aldrich). We captured images with FV1000 (Olympus). Each slide were chosen at least five randomly fields and analyzed in duplicate individual experiments.

### Western blotting

MLEC lysates were separated by 5 and 10% SDS-polyacrylamide gel electrophoresis, and then were transferred onto polyvinylidene difluoride membranes. The membranes were blocked with blocking buffer (LI-COR Biosciences, Lincoln, NE) for 1 h at room temperature and incubated with the anti-caspase-1 antibody (Abcam, Cambridge, MA) at 4 °C overnight. After washing, membranes were incubated with secondary antibodies (LI-COR Biosciences) for 1 h. Protein bands were visualized using the Odyssey System (LI-COR Biosciences).

### Statistical analysis

GraphPad Software was used to analyze data by two-tailed unpaired Student’s *t*-tests or one-way analysis of variance tests. No randomization was used in these studies. Data are presented as mean ± SEM. *P* < 0.05 was considered statistically significant (*P < 0.05; **P < 0.01).

## Electronic supplementary material

Supplementary figure legends

Supplementary figure 1

Supplementary figure 2
