# Supplementary material for: Group 2 innate lymphoid cells protect lung endothelial cells from pyroptosis in sepsis
Source: Cell Death Dis. 2018 Mar 6;9(3):369. doi: 10.1038/s41419-018-0412-5 (PMC5840374; doi:10.1038/s41419-018-0412-5)
Supplement: Supplementary file 1 — Supplementary figure legends [file 41419_2018_412_MOESM1_ESM.docx]

Supplementary Material

**Group 2 Innate Lymphoid Cells Protect Lung Endothelial Cells from Pyroptosis in Sepsis**

Dengming Lai, Jing Tang, Linsong Chen, Erica K. Fan, Melanie J. Scott, Yuehua Li, Timothy R. Billiar, Mark A. Wilson, Xiangming Fang*, Qiang Shu*, Jie Fan*

*** Correspondence:** X.F. (email: xmfang@zju.edu.cn), Q.S. (email: [shuqiang@zju.edu.cn](mailto:shuqiang@zju.edu.cn)) or J.F. (email: jif7@pitt.edu).

**Supplementary Figure 1**

**Supplementary Figure 1. Gating strategy for the identification of ILC2**

(**a**) Representative flow cytometry plots showing gating strategy to identify and quantify ILC2 in lung tissue. A lymphocyte gate was drawn (according to FSC and SSC properties) and doublets were excluded. CD45^+^ leucocytes were further gated into Lin^-^ and then ST2^+^CD90.2^+^.

Lin markers include B220, CD3, CD4, CD5, CD8α, CD11b, CD11c, CD19, Gr-1, TCRβ, Ter-119, γδTCR, NK1.1, FcεR1. (**b**) Expression of ILC2-identifying cell surface markers (Sca-1, KLRG1, CD25, CD127) on Lin^-^CD45^+^CD90.2^+^ST2^+^ lung ILC2 in WT mice after sham surgery (S: light blue line) and CLP (red line) compared to isotype controls (gray shaded).

**Supplementary Figure 2**

**Supplementary Figure 2. The expression of IL-9R on endothelial cell**

(**a**) Expression of IL-9R (red) on CD31^+^ MLEC cells from WT mice compared to isotype controls (gray shaded). Bar graph showing mean fluorescence intensity (MFI) of IL-9R compared with isotype control. Data shown mean ± SEM, n=3 mice/group. **P*<0.05, ***P*< 0.01, NS = not significant.
