# Supplementary figures and images for: Group 2 innate lymphoid cells protect lung endothelial cells from pyroptosis in sepsis
Source: Cell Death Dis. 2018 Mar 6;9(3):369. doi: 10.1038/s41419-018-0412-5 (PMC5840374; doi:10.1038/s41419-018-0412-5)

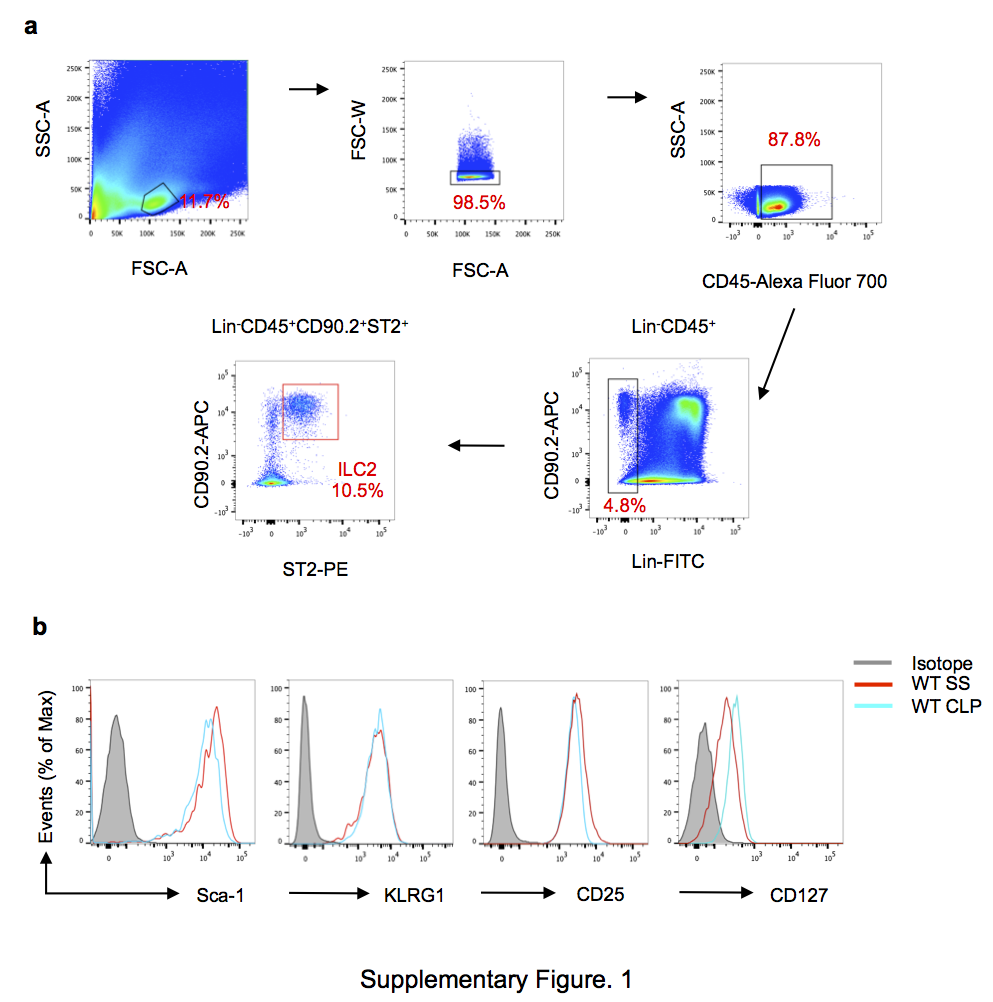

Supplement: Supplementary file 2 — Supplementary figure 1 [file 41419_2018_412_MOESM2_ESM.tif]

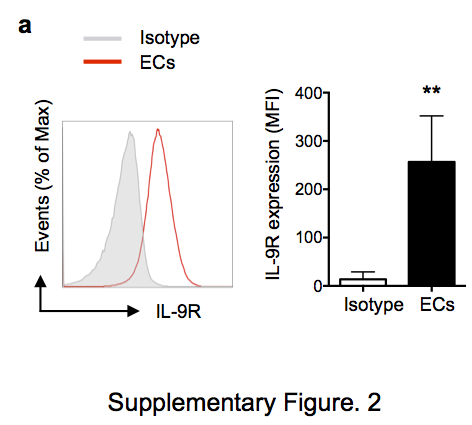

Supplement: Supplementary file 3 — Supplementary figure 2 [file 41419_2018_412_MOESM3_ESM.tif]
